# A Histone-Like Protein Induces Plasmid DNA to Form Liquid Crystals *in Vitro* and Gene Compaction *in Vivo*

**DOI:** 10.3390/ijms141223842

**Published:** 2013-12-06

**Authors:** Shiyong Sun, Mingxue Liu, Faqin Dong, Shenglan Fan, Yanchen Yao

**Affiliations:** 1 Key Laboratory of Solid Waste Treatment and Resource Recycle & Fundamental Science on Nuclear Waste and Environmental Security Laboratory, Southwest University of Science and Technology, Mianyang 621010, Sichuan, China; E-Mail: dragonlmx@126.com; 2 School of Environment and Resource, Southwest University of Science and Technology, Mianyang 621010, Sichuan, China; E-Mails: shenglanfan@163.com (S.F.); yyc930124@163.com (Y.Y.)

**Keywords:** liquid crystal, gene condensation, plasmid, histone-like protein, non-viral gene delivery

## Abstract

The liquid crystalline state is a universal phenomenon involving the formation of an ordered structure via a self-assembly process that has attracted attention from numerous scientists. In this study, the dinoflagellate histone-like protein HCcp3 is shown to induce super-coiled pUC18 plasmid DNA to enter a liquid crystalline state *in vitro*, and the role of HCcp3 in gene condensation *in vivo* is also presented. The plasmid DNA (pDNA)-HCcp3 complex formed birefringent spherical particles with a semi-crystalline selected area electronic diffraction (SAED) pattern. Circular dichroism (CD) titrations of pDNA and HCcp3 were performed. Without HCcp3, pUC18 showed the characteristic B conformation. As the HCcp3 concentration increased, the 273 nm band sharply shifted to 282 nm. When the HCcp3 concentration became high, the base pair (bp)/dimer ratio fell below 42/1, and the CD spectra of the pDNA-HCcp3 complexes became similar to that of dehydrated A-form DNA. Microscopy results showed that HCcp3 compacted the super-coiled gene into a condensed state and that inclusion bodies were formed. Our results indicated that HCcp3 has significant roles in gene condensation both *in vitro* and in histone-less eukaryotes *in vivo*. The present study indicates that HCcp3 has great potential for applications in non-viral gene delivery systems, where HCcp3 may compact genetic material to form liquid crystals.

## 1. Introduction

A liquid crystal is a type of material that displays physical and chemical characteristics between those of well-organized crystalline solids and free-flowing liquids. In the past decades, the basic properties and functions of liquid crystals have been intensively studied by biologists, chemists, physicists and material scientists [1,2]. At very high concentration in solution, DNA can spontaneously self-assemble into multiple liquid crystalline (LC) states *in vitro* [3]. Lyotropic LC DNA exists in mesophase states that have partially ordered crystalline organization, but the constituents still can flow and diffuse [4,5]. The formation of LC DNA is not only controlled by concentration but is also greatly influenced by the solvent environment. These LC mesophases can occur at low DNA concentrations in the presence of a monovalent salt due to the excluded volume effect [6]. The DNA length does not influence the characteristics of the LC phase for similar DNA concentrations and ionic conditions [3]. However, the length of the DNA influences the concentration required for a phase transition and the position of the phase boundary. The critical concentration for isotropic-anisotropic transition decreases as the DNA length increases [7]. Significantly lower critical concentrations are required for the formation of LC phases by super-coiled pDNA than by linear DNA [8,9].

In addition to DNA self-assembly, neutralization of the negative charges of DNA via counterion condensation can also form LC DNA [10]. In non-viral gene therapy applications, the formation of a reversible LC state of DNA is a crucial step that packages genes for the efficient transfer of undamaged DNA into eukaryotic cells [11]. Hydrophilic neutral polymers, such as polyethylene glycol (PEG) and polyvinylpyrrolidone (PVP), can induce DNA condensation in the presence of an adequate monovalent salt concentration through volume-exclusion effects [6]. The salt is necessary to screen the electrostatic repulsion forces of DNA and thus promote DNA condensation, which occurs between semiflexible DNA molecules and flexible neutral polymers [12,13]. The neutral polymer concentration required for DNA condensation decreases as the degree of polymerization or the salt concentration increases due to volume-exclusion and electrostatic repulsion effects. Poly-l-lysine (PLL) [14], polyamidoamine (PAMAM) [15] and polyethylenimine (PEI) [16] are the most widely used polycationic vectors for pDNA condensation in non-viral gene delivery systems. However, polymers that have too many positive charges in their amino groups will increase cell toxicity. Designing polycationic polymers with fewer charges to promote DNA condensation is an important way to optimize the gene transfection efficiency [17–21].

DNA can be tightly compacted into multiple condensation states by cationic counterions [22–24]. DNA condensation by counterions plays a critical role in biological functions [22]. In eukaryotic nuclei, multiple chromosomes are required to store genetic information, and each chromosome contains a large amount of DNA. DNA condensation is vital for packaging long-chain DNA (~2 m) into the cell nucleus (several microns in diameter) to store, transport and preserve genetic material [25]. In histone-less dinoflagellates, spermatozoa and some species of prokaryotes, DNA molecules are condensed to LC states *in vivo* [26]. Understanding the dynamic process of DNA condensation *in vitro* is an indirect way to study DNA packaging in an intracellular nucleus [27].

The histone-less dinoflagellates present a LC nucleus throughout their life cycle [28]. The nuclei of dinoflagellates contain a group of lysine-rich DNA-binding histone-like proteins (HLPs) that are associated with numerous genomes [28–31]. The function of HLPs may relate to stabilization of their distinctive chromosomal organization [32]. Four isoforms of *Crypthecodinium cohnii* histone-like proteins (HCc) were identified and termed HCcp1 to HCcp4 [33,34]. Our previous study suggested that HCcp3 is a DNA-binding and DNA-condensing protein that can induce linear DNA to enter a cholesteric LC state [29]. However, the mechanism of HCcp3-induced pDNA condensation is poorly understood. In the present report, the interaction between HCcp3 and pDNA was studied *in vitro*, and over-expressed HCcp3 in *Escherichia coli* was also studied to determine the roles of HCcp3 both *in vitro* and *in vivo*.

## 2. Results and Discussion

**Figure 1.**
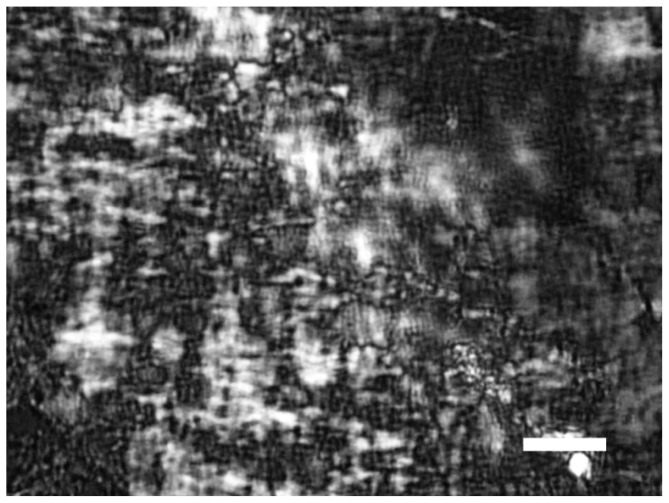
Polarization microscopy images of spherical particle-like pDNA-HCcp3 liquid crystalline condensates with a bp to dimer ratio of 20 to 1. The pDNA is the pUC18 plasmid. The birefringence (white and dark color) presented in figure indicated liquid crystalline phase. Scale bar in panel: 20 μm.

**Figure 2.**
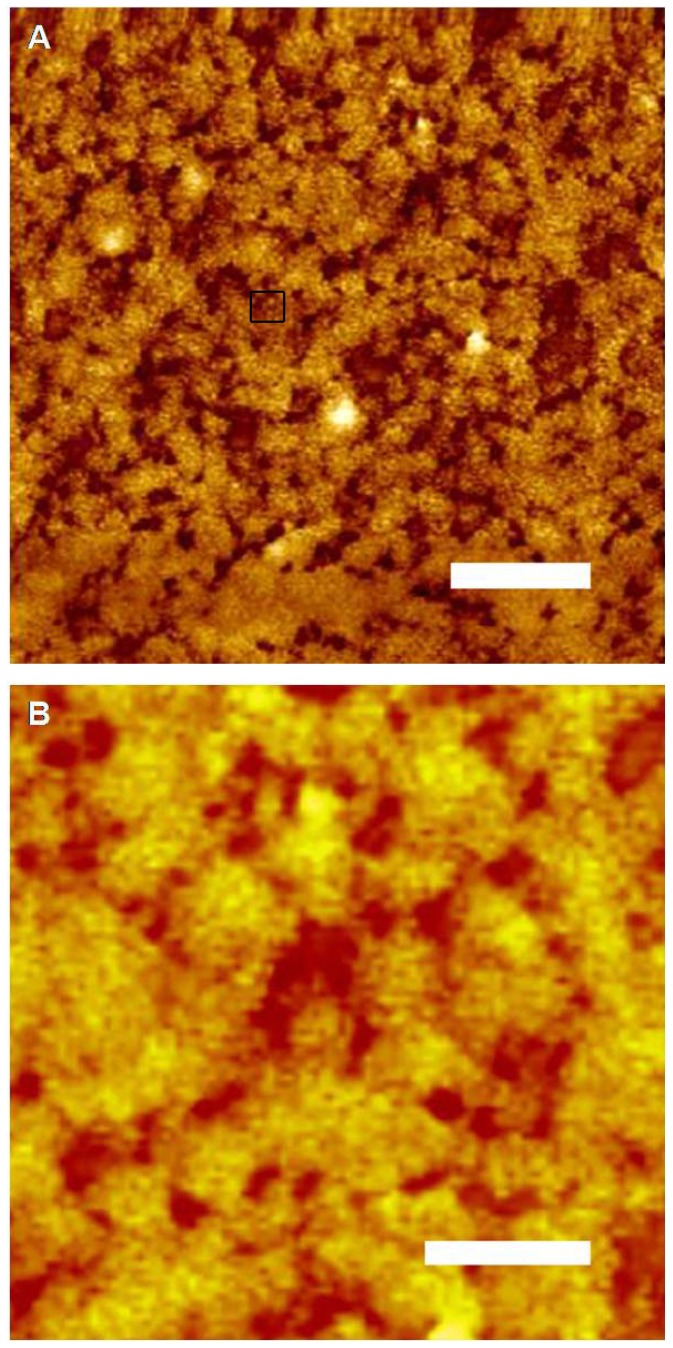
Atomic force microscopy micrographs of spherical pDNA-HCcp3 particles with a bp to dimer ratio of 20 to 1. (**B**) The magnified micrograph of selected area in (**A**). The The pDNA is the pUC18 plasmid. Scale bar in panel (**A)** 1 μm; Scale bar in panel (**B)** 200 nm.

**Figure 3.**
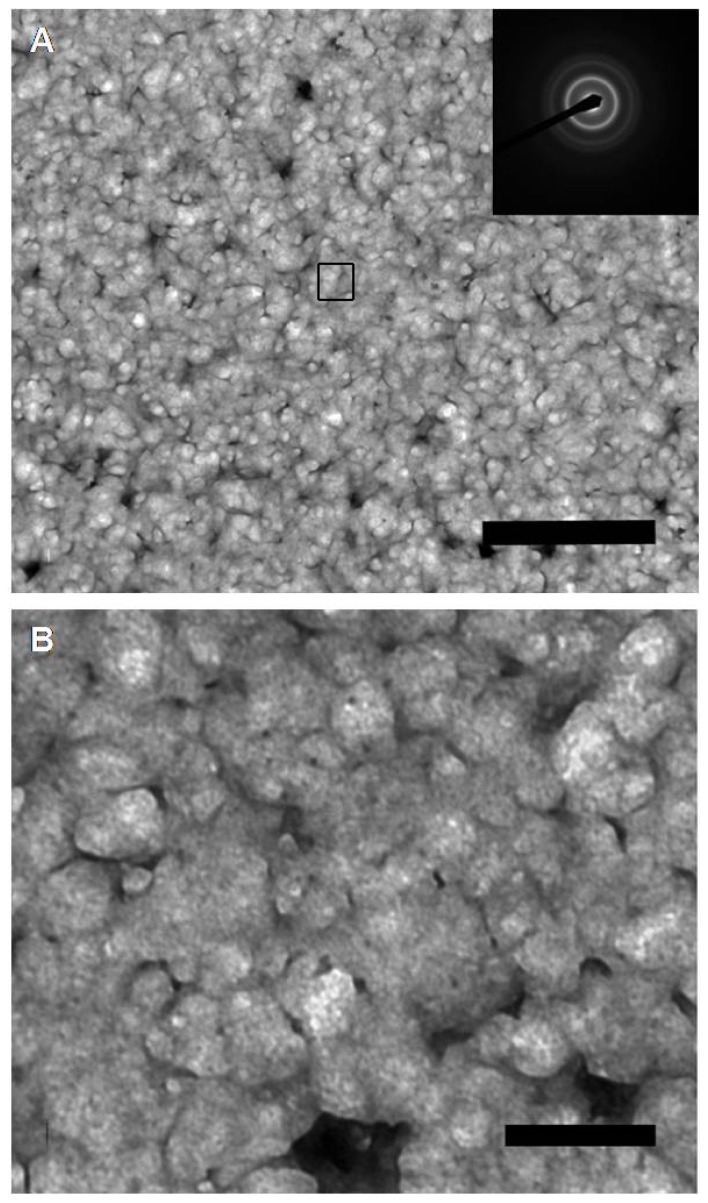
Transmission electron microscopy images of pDNA-HCcp3 The pDNA is the pUC18 plasmid, and the bp to dimer ratio is 20 to 1. (**B**) The magnified micrograph of selected area in (**A**). The inset in (**A**) is the selected area of the electron diffraction patterns showed several rings, indicating that the liquid crystalline particles of pDNA-HCcp3 complexes were not well crystallized. Scale bar in panel (**A)**: 1 μm; Scale bar in panel (**B)** 200 nm.

The pDNA-HCcp3 complexes showed various features depending on the amount of pDNA and HCcp3. At high HCcp3 concentrations, the pDNA-HCcp3 showed birefringent textures (Figure 1). However, the birefringent domains were inhomogeneously distributed. The pDNA-HCcp3 complexes appeared to form spherical particles. The results of AFM with nanoscale resolution showed that the pDNA-HCcp3 complexes formed sphere-like nanoparticles (Figure 2). These results were also confirmed by TEM (Figure 3). The SAED pattern, shown in the insert in Figure 3A, shows that the complexes are mesophase crystals.

**Figure 4.**
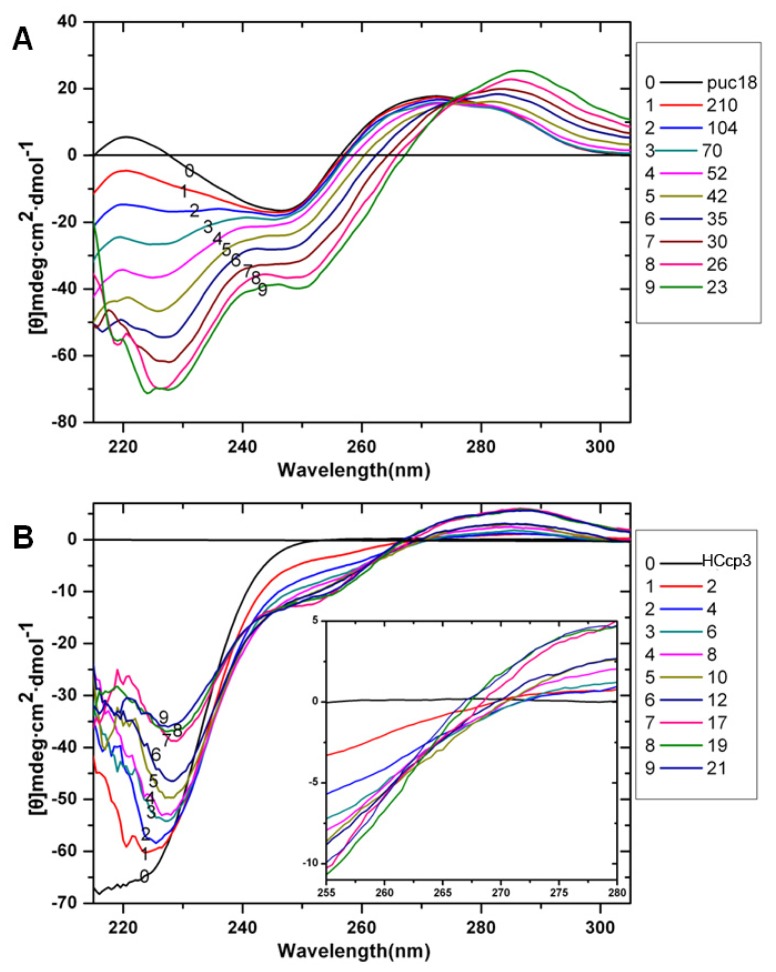
Circular dichroism spectra of HCcp3 titrated with pDNA at various bp to dimer ratios. (**A**) 0.1 mg·mL^−1^ HCcp3 titrated into 50 μg·mL^−1^ pUC18 plasmid; (**B**) 100 μg·mL^−1^ pUC18 plasmid titrated into 0.1 mg·mL^−1^ HCcp3. The inset of (**B**) shows the enlarged region from 255 to 280 nm of (**B**). Data collected from 320 to 200 nm. The bp to dimer ratio of every titration is indicated in the images.

CD is the most common and convenient spectroscopic technique for detecting changes in DNA secondary structure [35]. Therefore, CD was utilized in our study to determine the pDNA conformations throughout the HCcp3-induced condensation process. Using a fixed concentration of 51.2 μg per mL, the binding of HCcp3 to pDNA was monitored by CD (Figure 4). The CD spectra of the circular pDNA showed the characteristic B form. As the HCcp3 concentration increased, the 273 nm band sharply shifted to 282 nm without changes to the negative band. When the HCcp3 concentration became high, the bp/dimer ratio fell below 42/1, and the CD spectra of the pDNA-HCcp3 complexes indicated a conformation that was similar of that of A-form DNA [36].

In the cholesteric LC phase, HCcp3 can cause semiflexible linear DNA molecules to become aligned parallel to each other and to compact into multiple ordered bundles [29]. In contrast, the more rigid super-coiled circular pDNA will be compacted together into plasmids in the presence of HCcp3 (Figures 2B and 3B).

The highly negatively charged linear DNA chain is not a rigid rod in salt solution; instead, the DNA chain adopts a dynamic random coil conformation with a persistence length of approximately 50 nm [3,37]. Under physiological conditions, linear DNA most commonly exists in the B form. However, its secondary conformation can be affected by the surrounding environment (e.g., counterion, hydration, solvent and salt effects) [38]. Analysis of the conformations of DNA while it is condensed by counterions suggests that the B form is the most resistant to nonspecific aggregation because of its groove widths. The B-form conformation is more stable in high hydration conditions, and the A form is more stable in conditions of low hydration and high ionic strength. Numerous DNA-protein complexes also underwent the B-A DNA transition upon binding by proteins [39,40]. Dehydration plays an important role in this B-A transition because dehydration causes rearrangement of the water molecules between DNA duplexes [41]. Consequently, changes in the intensity of the CD band near 273 nm have been associated with the hydration-induced alteration of the pDNA double helix [42,43]. To some extent, the transition from B to A can be viewed as a dehydration process that closes the major groove of the B form [44,45]. The binding of HCcp3 to the pDNA surface results in particularly strong electrostatic forces that are associated with the torsional stress from the superhelicity of the plasmid, which slightly unwinds the super-coiled pDNA and induces the B-A transition to close the major groove of the B form. The strength of the B-A transition forces and the rapid aggregation of super-coiled circular pDNAs by intermolecular electrostatic forces may counteract HCcp3 alignment forces and induce particle-like aggregation of the pDNA-HCcp3 (Figures 2 and 3).

DNA condensation by cobalt hexamine (Co(NH_3_)_6_^3+^) was intensively studied [36,41]. DNA binding by Co(NH_3_)_6_^3+^ is accompanied by a significant dehydration process. At high alcohol concentrations, condensation of partly dehydrated pDNA by Co(NH_3_)_6_^3+^ was detected along with a B-A pDNA conformational transition [36]. These preliminary results suggested that the HCcp3-induced transition of pDNA to the LC state *in vitro* may result from a DNA condensing mechanism similar to that of cobalt hexamine, polyamine and some DNA-binding proteins [39,46]. In a manner similar to those condensing mechanisms, HCcp3 may bind to the major groove of B-form pDNA and induce a B-A conformational transition upon completion of the condensation process.

In the absence of counterions in the groove, DNA favors the B form, where as DNA prefers the A form when counterions are located in the groove [38]. DNA condensing agents, including cobalt hexamine, the polycations spermidine and spermine and basic nuclear proteins, are known to induce DNA condensation that is accompanied by a B to A or B to Z conformational transition [36,41]. Many studies of the condensation of DNA by proteins revealed that the B to A transition occurs once DNA binds to proteins [38]. The mechanism of the DNA conformational alterations is still debated. The roles of counterions in DNA conformational alterations are poorly understood. A growing viewpoint with experimental support is that the system of electrostatic free energy gain, especially in the presence of counterions, is very important for inducing the B to A transition [38].

Most common eukaryotic genomes are packaged by core histones or protamines [47,48]. One of the most abundant nucleoid-associated histone-like architectural proteins, HU (“H” for histone-like and “U” for U93), is essential for bacterial nucleoid condensation. HU is also involved in many important genetic processes, including transposition, recombination and DNA repair [48]. HU is a nonspecific DNA-binding protein that can bend DNA, but HU alone cannot compact DNA into a condensate [48,49]. HU can control which morphology (e.g., toroid or rod) is adopted by DNA condensates upon the addition of macromolecular crowding agents, such as PEG or spermidine [50]. Amino acid sequence analysis revealed that the homology between HCcp3 and the prokaryotic HU was greater than that between HCcp3 and the eukaryotic histone H1 [34]. Our earlier study of the DNA-binding properties of HCcp3 demonstrated the functional difference between HU and HCcp3 [32]. Therefore, interpretations based on our results suggest that HCcp3 not only is an efficient condensing pDNA agent similar to universal basic proteins such as HU but also can help to compact pDNA-HCcp3 condensates for assembly into LC nano-spheres.

**Figure 5.**
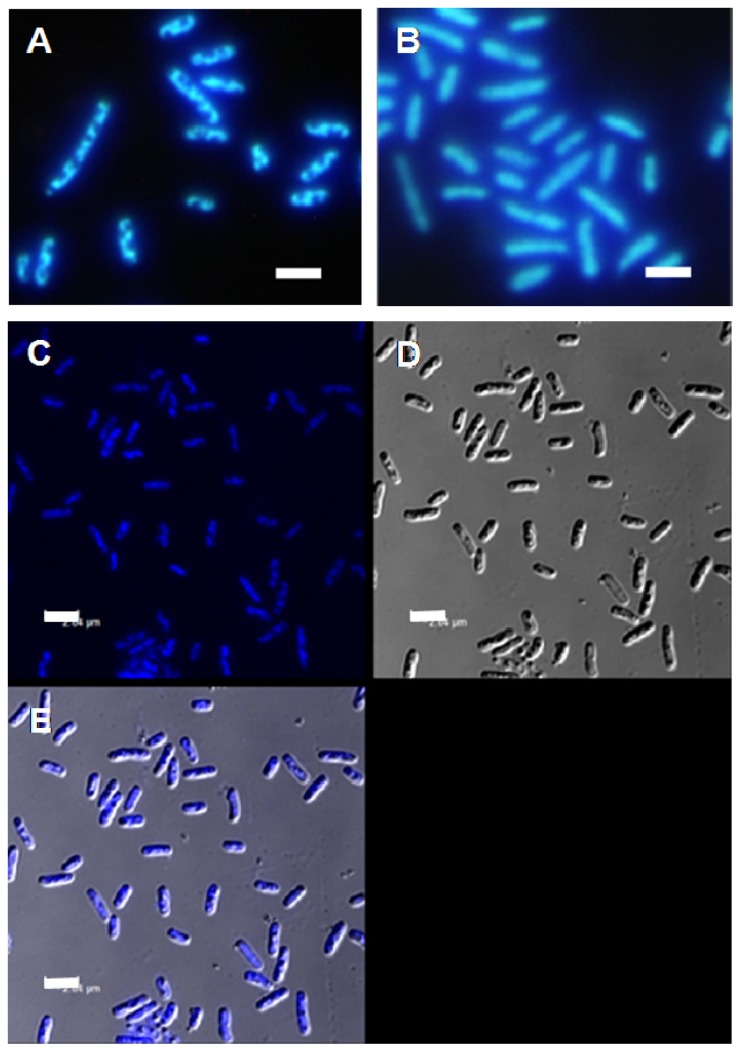
Fluorescence microscopy and confocal laser scanning microscopy images of DAPI-stained *E. coli*. (**A**) Over-expressed HCcp3 in *E. coli*; (**B**) The control *E. coli* without HCcp3 expression; (**C**–**E**) Over-expressed HCcp3 in *E. coli* observed using a Confocal Laser Scanning microscope; (**C**) fluorescent image of DAPI-stained *E. coli*; (**D**) Bright field image of DAPI-stained *E. coli*; (**E**) the merged images of (**C**) and (**D**). Scale bar: 2 μm.

**Figure 6.**
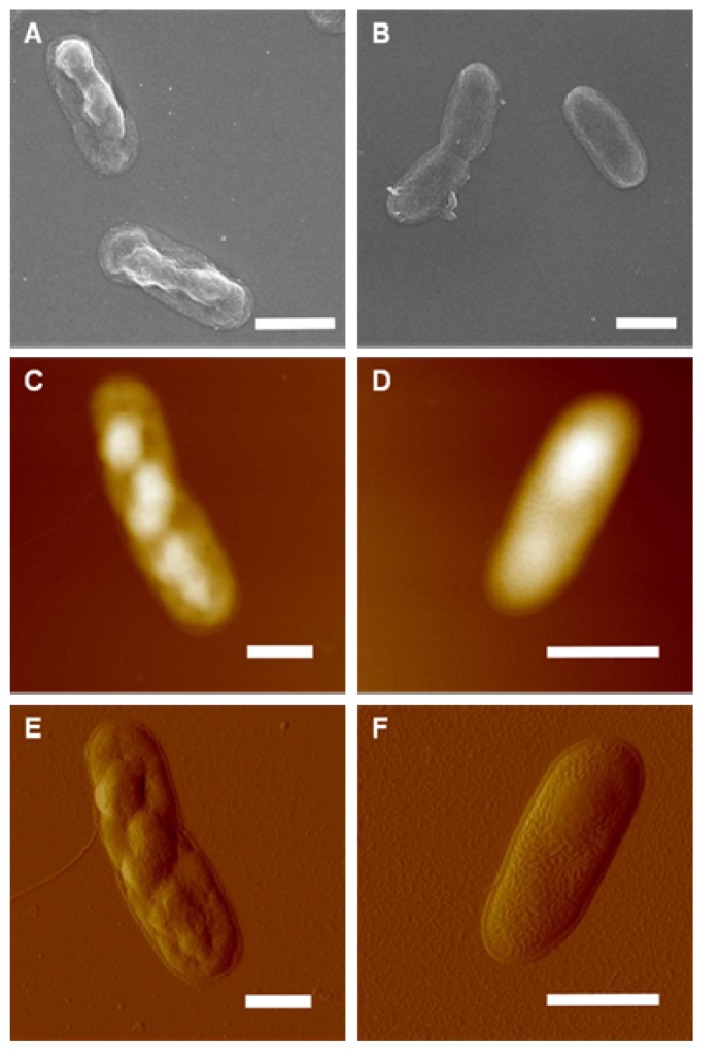
*E. coli* cells that over-expressed HCcp3 and control cells without HCcp3 expression. (**A**,**C**,**E**) Cells that over-expressed HCcp3; (**B**,**D**,**F**) Control cells; (**A**,**B**) Scanning electron microscopy images; (**C**–**F**) Atomic force microscopy images. Scale bar 1 μm.

**Figure 7.**
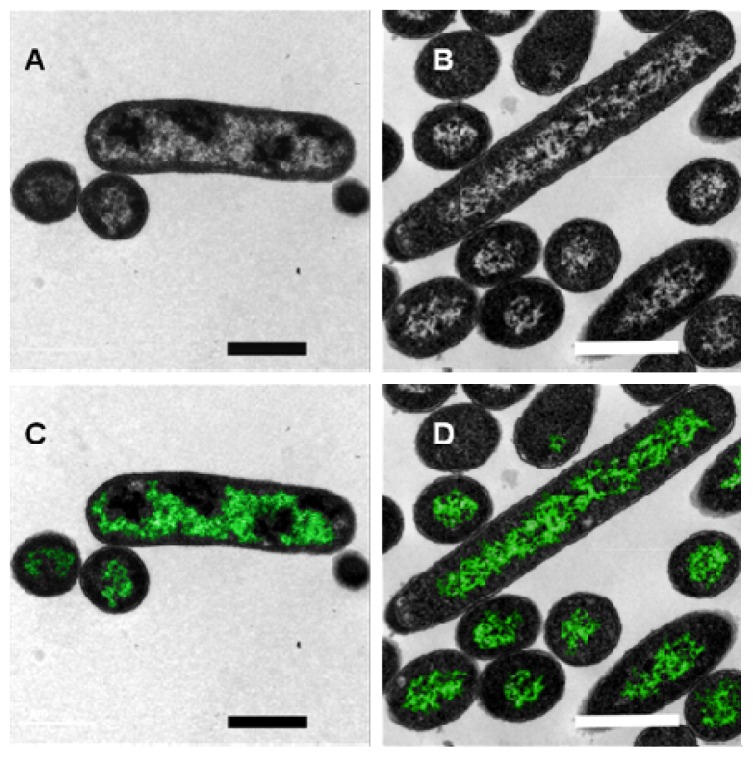
Transmission electronic microscopy images of *E. coli.* (**A**,**C**) Cells that over-expressed HCcp3; (**B**,**D**) Control cells without HCcp3 expression; (**C**,**D**) The false green color represents the nucleoid distribution in the cells. Scale bar: 1 μm.

**Figure 8.**
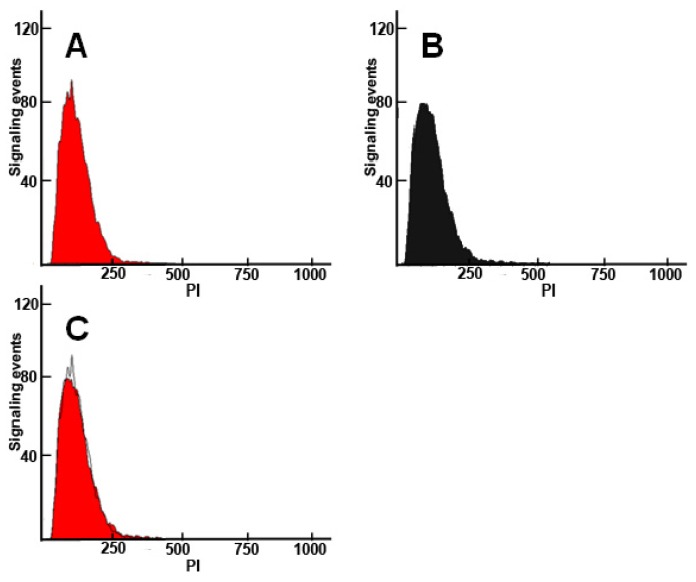
Flow cytograms of PI-stained *E. coli*. (**A**) Cells that over-expressed HCcp3; (**B**) Control cells without HCcp3 expression; (**C**) Overlay of the cytograms in (**A**) and (**B**).

To better evaluate the role of HCcp3 in pDNA condensation, the over-expressed HCcp3 and control *E. coli* cells were observed using multiple microscopy techniques. As shown in Figure 5, the nucleoids present a helical-like structure in the bacteria that over-expressed HCcp3, whereas the eukaryotes adopted a chromosomal structure. The nucleoids are homogenously distributed in the control cell, which did not express HCcp3 (Figure 5B). The results of the SEM and AFM studies show that the nucleoids form inclusion bodies in the *E. coli* that over-expressed HCcp3; this finding demonstrates the distinct role of HCcp3 in gene condensation *in vivo* (Figure 6). The TEM observations of the ultrathin sections of the *E. coli* that over-expressed HCcp3 and the control *E. coli* confirmed the helical-like distribution of nucleoids in the *E. coli* that over-expressed HCcp3 (Figure 7). However, the DNA content was similar in the cells that over-expressed HCcp3 and the control *E. coli*, which present similar fluorescence intensities in flow cytograms (Figure 8).

The nucleoid of *E. coli* occupies less than 25% of the cell volume and is distributed in the form of bodies within the living cell [51]. The DNA of the bacterial nucleoid is estimated to have a local concentration of ~50 to 100 mg·mL^−1^ with crowded macromolecular concentrations of 300 mg·mL^−1^[52–54]. There is no single protein or group of proteins that plays a predominant role in compacting pDNA into the nucleoid, although histone-like proteins such as HU have important contributions to pDNA condensation in bacteria [51].

Some reports suggested that super-coiling, cations, polyamines and histone-like proteins most likely contribute to the cellular compaction of nucleoids *in vivo* [52,54]. However, it is unclear how important each of these factors is in such nucleoid condensation. The nucleoids of the *E. coli* that over-expressed HCcp3 adopted a helical-like structure, indicating that HCcp3 plays an important role in gene condensation *in vivo* (Figures 5–7). Helical folding is the favored form of biological fibers *in vivo* due to the overlapping of the excluded volumes generated by the helix; thus, entropic attraction can stabilize such a structure [53,55].

## 3. Materials and Methods

### 3.1. Over-Expression and Purification of Recombinant HCcp3

DNA fragments encoding HCcp3 (GenBank accession: AY128510.1) were amplified by PCR and digested by the restriction enzymes BamHI and HindIII. Recombinant HCcp3 was fused with *N*-terminal 6× His-tagged polypeptides and expressed in *E. coli* SG13009. The recombinant HCcp3 was over-expressed and purified using a QIA expressionist protein expression and purification system (Qiagen Corporation, Boston, MA, USA) under denaturing conditions. The recombinant HCcp3 was further purified by a Bio-scale Mini UNO sphere S cartridge using Biological LPC (Bio-Rad, Alfred Nobel Drive Hercules, CA, USA). The purified recombinant HCcp3 was stored in 100 mM NaCl at −20 °C for experimental use. The protein concentrations were determined by a protein assay kit (Bio-Rad) using BSA as a standard protein.

### 3.2. Preparation of pDNA

The pUC18 plasmid (2686 bp) that was used as the pDNA in this paper was transformed into *E. coli* JM109 by the heat-shock method. The bacteria from a single transformed colony were cultured in ATCC 1065 LB medium with 100 mg·mL^−1^ ampicillin for 14 h at 37 °C under continuous shaking. The pDNA was purified by a pDNA purification kit (NucleoBond, Bethlehem, PA, USA). The purified pDNA was dialyzed against 100 mM NaCl. The integrity of the plasmid was checked with a 1% agarose gel in 1× TAE buffer with restriction endonuclease digestion. The gel electrophoresis showed that approximately 95% of the plasmid was in the closed circular form.

### 3.3. Atomic Force Microscopy

Quantitative determination of the ‘bp/dimer ratios’ can be found our previous work [32]. 100 μL samples of the pDNA-HCcp3 complexes were prepared for AFM observation by mixing HCcp3 and DNA at a bp/dimer ratio of 20/1 and incubating the complexes for 10 min at room temperature. A 10 μL droplet of the DNA-HCcp3 complexes was deposited onto the surface of a glass coverslip or a freshly cleaved mica substrate pre-treated with 50 mM MgCl_2_ for 5 min. For the tapping mode in air (Veeco NanoScope V, Plainview, NY, USA), the substrate was rapidly rinsed several times with ultrapure water (Millipore, Bilerica, MA, USA) and dried under nitrogen.

The *E. coli* for AFM were suspended in 1 × PBS buffer and rinsed several times. For tapping mode in air (Veeco NanoScope V, Plainview, NY, USA), the glass coverslip or freshly cleaved mica was rapidly rinsed several times with ultrapure water (Millipore, Bilerica, MA, USA) and dried under nitrogen. The images were flattened and analyzed using the software provided by the manufacturer.

### 3.4. Fluorescence Microscopy

The *E. coli* were suspended in 1 × PBS buffer and prefixed using 2% freshly prepared paraformaldehyde (PFA). The fixed bacteria were stained by 50 μg·mL^−1^ DAPI and sealed between a glass slide and a coverslip by Cytoseal 60 (Thermoscientific, Boston, MA, USA). The samples were observed using an inverted Leica SP5 confocal laser scanning microscope (CLSM, Leica, Wetzlar, Germany) or an ordinary fluorescence microscope.

### 3.5. Electron Microscopy

The *E. coli* were suspended in 1 × PBS buffer and prefixed using 2% freshly prepared paraformaldehyde (PFA), followed by post fixing with 2% osmium tetraoxide (OsO_4_) at room temperature for 2 h. The fixed *E. coli* were dehydrated by a series of 10%–100% graded ethanol. For transmission electronic microscopy (TEM; JEOL, Tokyo, Japan), the *E. coli* were embedded in Spurr’s epoxy resin and then cut into ultrathin sections. The ultrathin sections were mounted on carbon-coated copper grids and double stained with 1.5% lead citrate and 2% uranyl acetate for 15 min each. For scanning electronic microscopy (SEM; JEOL, Tokyo, Japan), the dehydrated bacteria were mounted on a silica wafer, and the electronic conductivity was enhanced by coating the sample with a thin layer of gold.

### 3.6. Polarization Microscopy

The samples for polarization microscopy (PM) were prepared by mixing 0.1 mg·mL^−1^ pDNA with 1 mg·mL^−1^ HCcp3 with bp/dimer ratio of of 20 to 1; the mixtures were then incubated for 10 min in 1.5 mL tubes (Eppendorf, Hauppauge, NY, USA) on ice. The samples were then collected by centrifugation for 15 min at 16.1 k × rcf at 4 °C (Eppendorf, Hauppauge, NY, USA). The pellets containing the pDNA-HCcp3 complexes were sealed between a glass slide and a coverslip using Cytoseal 60 (Thermo Scientific, Boston, MA, USA) to prevent dehydration. The samples were observed under an Olympus BX51 polarization microscope (Olympus, Tokyo, Japan) with 40× and 100× (oil immersion) objectives at ambient temperature.

### 3.7. Circular Dichroism

Circular dichroism (CD) spectra of the pDNA and HCcp3 titration experiments were recorded on a J-815 spectropolarimeter (Jasco, Tokyo, Japan) at room temperature. The CD spectra were expressed as molar ellipticity, [θ], (mdeg·cm^2^·dmol^−1^). The spectra were acquired in a 10 mm path length rectangular quartz cuvette (DNA concentration below 0.5 mg·mL^−1^) or 0.1 mm path length circular quartz cell (DNA concentration above 0.5 mg·mL^−1^). For each titration, the CD spectrum was obtained by averaging 3 successive accumulations at a scan speed of 50 nm/min and a wavelength range from 320 to 200 nm. The baseline was corrected by subtracting the buffer spectrum.

## 4. Conclusions

Various independent techniques were used to study the interactions of pDNA and HCcp3 both *in vitro* and *in vivo*. All results consistently demonstrated that pDNA can be compacted by the histone-like protein HCcp3 to form a liquid crystal; during this process, the DNA undergoes a B to A conformation transition via the universal dehydration process. Our results showed that the predominant roles of histone-like proteins in pDNA are charge neutralization and the assembly of liquid crystalline states *in vitro* and compaction for gene condensation with the formation of inclusion bodies *in vivo*. The present study also indicated that the compaction of genetic material to liquid crystals by histone-like proteins might be a feasible method for non-viral gene delivery systems to transform genetic material that is generated *in vitro* into eukaryotes *in vivo*.
